# Treatment plan comparison for irradiation of multiple brain metastases with hippocampal avoidance whole brain radiotherapy and simultaneous integrated boost using the Varian Halcyon and the Elekta Synergy platforms

**DOI:** 10.1186/s13014-022-02156-6

**Published:** 2022-11-21

**Authors:** Johannes Kraft, Stefan Weick, Kathrin Breuer, Paul Lutyj, Klaus Bratengeier, Florian Exner, Anne Richter, Jörg Tamihardja, Dominik Lisowski, Bülent Polat, Michael Flentje

**Affiliations:** Department of Radiation Oncology, University Hospital Wuerzburg, Josef-Schneider-Straße 11, 97080 Wuerzburg, Germany

## Introduction

Around 20–40% of all cancer patients develop brain metastases during their course of disease which is associated with significant morbidity and mortality [1]. Brain metastases mostly occur in patients with lung cancer, breast cancer and melanoma [2]. The incidence generally slightly increased over the last years and especially the number of patients with multiple brain metastases is increasing [3]. The treatment of limited brain metastases is well defined in clinical guidelines and comprises upfront radiosurgery, resection followed by adjuvant radiosurgery/fractionated stereotactic radiotherapy or systemic therapy in cancer with a targetable driver mutation or excellent response to immunotherapy such as melanoma [4]. In contrast, the treatment of patients with multiple brain metastases is more challenging and a clear recommendation is not given within the guidelines [3]. There are two competing major radiotherapy policies for the treatment of multiple brain metastases—whole brain irradiation (WBRT) on the one hand and radiosurgery (SRS) on the other hand. Stereotactic radiotherapy or radiosurgery approaches apply a high dose to the tumor with a steep dose fall off to the surrounding tissue to spare as much as possible healthy brain tissue. Nevertheless, microscopic tumor spread within the brain is more likely with multiple brain metastases which can be drawn from a higher risk of loco-regional (distant brain) failure with SRS in multiple brain metastases [5, 6]. Whole-brain radiotherapy concepts have not been displaced by stereotactic radiotherapy in multiple brain metastases and yield the benefit of treating microscopic metastatic spread. However, the irradiation of the whole brain comes along with a detrimental neurocognitive decline with damaged neural stem cells within the hippocampal being the most relevant cause [7, 8]. Therefore hippocampal radiotherapy techniques have been introduced to spare the stem cells within the hippocampal area [9]. Hippocampus avoidance whole-brain irradiation (HA-WBRT) with simultaneous integrated boost (SIB) is the most sophisticated concept within whole-brain irradiation concepts, where in addition to hippocampal sparing and prophylactic dose to the healthy brain, dose escalation in the gross tumor volume is pursued to improve local control [10].

Several randomized trials aim to clarify the optimal treatment strategy for patients with multiple brain metastases, with the majority of these trials still recruiting. To date, only few results have been published. A dutch trial randomizing patients with 4–10 brain metastases to SRS or WBRT showed higher one year survival rates with SRS (57%) compared to WBRT (31%) with worse brain salvage-free survival rate with SRS, although the trial did not show significance due to poor accrual and premature closure [11]. Results of another trial randomizing patients with 4–15 non-melanoma brain metastases to SRS or conventional WBRT favor the use of SRS, with less cognitive decline and equivalent overall survival [12]. However, neither hippocampal avoidance (HA) nor dose escalation techniques within metastases (e.g. simultaneous integrated boost, SIB) were used in the previously mentioned studies. The protective effect of HA-WBRT on neurocognition initially observed in RTOG 0933 and confirmed with several recent randomized trials has shifted the main focus to the comparison of HA-WBRT versus SRS, where data on a randomized comparison is still lacking [13–15]. Results of several trials randomizing patients with multiple brain metastases to HA-WBRT or SRS are awaited (NCT03550391, NCT03075072, NCT04277403, NCT03075072). Considering the limited data and numerous outstanding trials, up to now, there is no high evidence to justify the use of HA-WBRT(+/-)SIB over SRS alone or WBRT.

Recently Varians new O-ring Linear Accelerator (Halcyon) has been introduced in clinical practice. The Halcyon utilizes a 6 MV flattening filter-free (FFF) beam with a novel double stack multi-leaf collimator (MLC) which promises high delivery efficiency and enables high modulation. The application of the machine focuses in particular on extracerebral radiotherapy. Nevertheless, with the widespread adoption of this new linear accelerator the question arises whether intracerebral HA-WBRT+SIB could be applied and if one can expand its applicability. For this purpose, we analyzed Pinnacle (Philips Radiation Oncology Systems) treatment plans of HA-WBRT+SIB for irradiation at a Elekta Synergy Agility linac and compared them with re-planned treatment plans, optimized with Eclipse for irradiation on Halcyon-Linac (Varian Medical Systems, Palo Alto, CA). Treatment planning was made according to the experimental arm of the german HIPPORAD study, a prospective, randomized, phase II trial (DRKS00004598) evaluating the impact of hippocampal sparing on the neurocognitive function (primary endpoint) with HA-WBRT + SIB versus WBRT + SIB in patients with multiple brain metastases [16]. Here, 30 Gy in 12 fractions are prescribed for the healthy brain with 51 Gy in 12 fractions as a simultaneous integrated boost (SIB) to the metastases. In the experimental arm a simultaneous integrated protection (SIP) to both hippocampi is applied. Inclusion criteria include patients with 4 to 10 brain metastases (> 5 mm diameter), Recursive partitioning analysis (RPA) classification I or II and no metastases within the hippocampus or in a distance of 7 mm to the hippocampus.

## Methods

### Patient selection

For the present study patients with a diagnosis of brain metastases treated with radiotherapy in our institute between January 2015 and December 2020 were screened. Patients with multiple brain metastases (> 4) treated with HA-WBRT + SIB were enrolled in this study. Patients with brainstem metastases were excluded for better comparability, as a reduced simultaneous integrated boost dose is usually used in those cases. All patients had clinically accepted treatment plans for irradiation at a C-arm Elekta Synergy Agility linac and were planned according to the HIPPORAD study protocol. Data on patient characteristics, primary tumor, number and size of brain metastases and treatment procedures was retrospectively collected. All patients signed an informed consent at hospital admission for retrospective data analysis.

### Contouring and dose prescription

Contouring and dose prescription was made analog to the study protocol of the phase II prospective randomized multicenter HIPPORAD trial [11]. For target and organ at risk delineation T1-weighted MRI sequence with intravenous application of a Gadolinium contrast medium has been co-registered with contrast enhanced computed tomography with 1–2 mm slice thickness in planning position. Patients have been immobilized with thermoplastic masks. Gross tumor volume for each metastasis (summed up as GTVmetastases) was contoured according to the visible metastasis on contrast enhanced T1-weighted MRI. Planning tumor volume for metastases (PTVmetastases) was created by a 1 mm 3-dimensional margin expansion of the GTVmetastases. Clinical target volume (CTV) for whole brain (CTVwholebrain) was defined as the whole-brain parenchyma. Bilateral hippocampal contours were delineated on the co-registered MRI according to the contouring atlas for RTOG 0933. A hippocampal avoidance region (HAR) was added with a 7 mm margin around the hippocampus. Planning target volume (PTV) for wholebrain (PTVwholebrain), has been defined as the CTVwholebrain with an additional margin of 5 mm and excluding the PTVmetastases and the hippocampal avoidance regions (HAR). Several organs at risk have been added according to internal standards also being part of the study protocol (eyes, lenses, optical nerves, chiasm, inner ears and brainstem).

The prescribed dose was 30 Gy in 12 fractions with 2.5 Gy per fraction to the PTVwholebrain and 51 Gy in 12 fractions with 4.25 Gy per fraction as a simultaneously integrated boost (SIB) to individual brain metastases. The dose was prescribed such that 95% of the PTV was covered by the prescription dose.

### Treatment planning and VMAT optimization

Treatment planning was performed with Pinnacle version 16.2 (Philips Radiation Oncology Systems) for treatment at a Synergy Agility linear accelerator and with Varian Eclipse Treatment Planning Software (version 15.6, Varian Medical Systems, Palo Alto, CA, USA) for treatment at Varians Halcyon. Treatment planning was made according to the requirements of the HIPPORAD study protocol. Only one optimization cycle was performed with individual optimization of objectives and weights to achieve the planning goals. An overview of treatment planning parameters and dose constraints of OARs is given in Table 2. All treatment plans were created by an experienced medical physicist. Plan optimization in Pinnacle is gradient-based. For plan optimization in Pinnacle its Auto-Planning module version 16.2.1 was used to achieve the planning goals, whereas Eclipse utilized the Photon Optimizer algorithm version 15.6.06 based on individual optimization objectives and weights (without knowledge-based solution). Pinnacle utilized the collapsed cone convolution algorithm and a grid size of 0.2 cm for dose distribution computation, while Acuros External Beam version 15.6.06 (AcurosXB) with a grid size of 0.25 cm was used for dose distribution computation on the Eclipse TPS.

### Synergy agility linear accelerator

The C-Arm linear accelerator Synergy with an Agility head (Elekta AB) offers a maximum dose rate of 500 MUs per minute with a flattened beam profile, a maximum field size of 40 × 40 cm and interdigitating leaf pairs with a projected width of 5 mm at isocenter. In addition, kV‐CBCT and a variable photon beam energy of 6 MV, 10 MV, and 18 MV are available.

### Halcyon linear accelerator

The Halcyon consists of a ring-based linear accelerator with a 6 MV FFF photon beam, with a maximum dose rate of 800 monitor units (MUs) per minute and a maximum field size of 28 × 28 cm per isocenter. Halcyon 2.0 offers MV‐based cone‐beam computed tomography (MV‐CBCT) as well as kV‐based cone‐beam computed tomography (kV‐CBCT). The Halcyon has dual‐layer stacked–staggered MLCs with 10 mm leaf width. The gantry speed reaches up to four rotations per minute (RPM) and treatment is delivered at a maximum of 2 RPM. CBCT‐imaging prior to each treatment session is mandatory using the Halcyon system, because there are no optical distance indicators or lasers available at treatment isocenter.

### Beam configuration

**Fig. 1:**
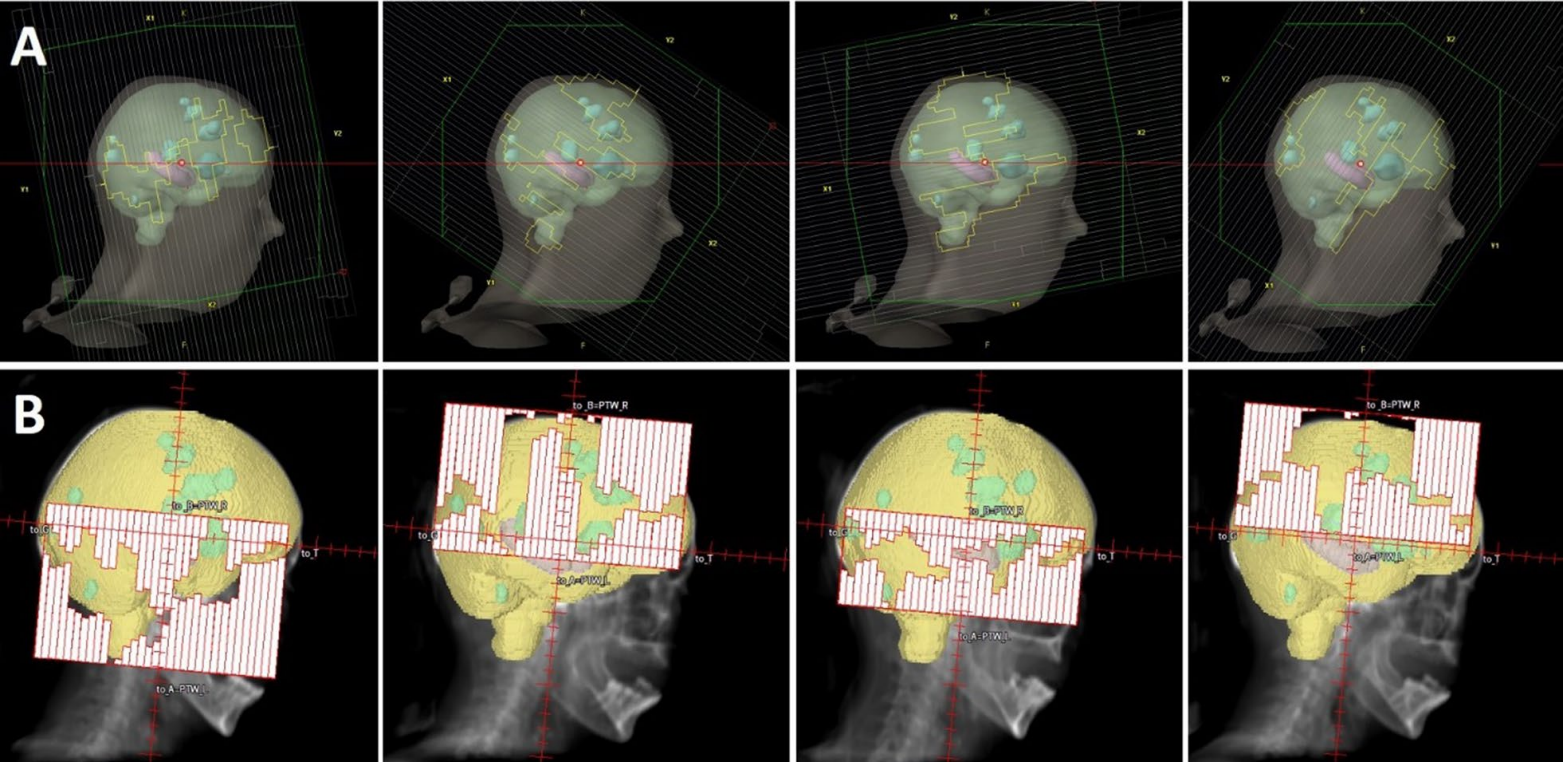
Example of treatment fields for HA-WBRT + SIB. Colored structures: PTVwholebrain, PTVmetastases and HAR. **A** Halcyon Beams Eye view from 270° illustrating the four beams with different collimator setting of 281°, 326°, 11° and 56°. **B** Pinnacle Beams Eye view from 270° showing 4 half beams (each beam splitted at the hippocampal level). Two arcs covered the cranial part of the brain and two arcs covered the caudal part of the brain

Two beams with two full rotations (Arcs) each, where each beam is splitted at the level of the hippocampi, were used to create plans for the Synergy linac in Pinnacle. Therefore, one beam covers the cranial part of the brain and the second beam the caudal part. Collimator setting was 85°. Beams eye views can be seen in Fig. 1. Four beams with four full rotations and a collimator setting of 281°, 326°, 11° and 56° were used to create plans for the Halcyon in Eclipse.

### Treatment plan evaluation and statistics

Treatment plans were evaluated on target dose and coverage, dose to the hippocampus and dose to organs at risk. Pinnacle plans were imported into Eclipse Treatment Planning System (TPS) (version 15.6, Varian Medical Systems, Palo Alto, CA, USA) and hereafter dose volume histograms (DVHs) of all 24 treatment plans were exported in text format, imported into R (version 3.3.2., R Foundation for Statistical Computing, Vienna, Austria) and processed using DVH metrics library (https://cran.r-project.org/web/packages/DVHmetrics/index.html) to calculate the corresponding values of the study protocol. Matched-pairs t-tests were applied for the comparison of Synergy and Halcyon Dose-Volume-Histogram parameters for normally distributed parameters according to the Shapiro-Wilk test if not indicated otherwise. In any case of non-normally distributed parameters Wilcoxon matched-pairs rank tests were applied instead. Statistical significance was declared in case of a two sided *p*-value < 0.05. Statistical analysis was conducted using (GraphPad Prism, version 6.00, GraphPad Software, San Diego, California, USA “www.graphpad.com”). Quantitative values are expressed as mean ± standard deviation.

In terms of clinical suitability, all treatment plans were evaluated for adherence to the HIPPORAD study protocol requirements. Minor and major protocol deviations are reported in Table 2. Homogeneity index, conformity index and gradient index were calculated. To account for the effects of the dose gradients, the homogeneity index was calculated on scaled-down structures (PTVwholebrain minus 5 mm and PTVmetastases minus 2 mm). According to ICRU83, Homogeneity index (HI) was defined as D2% minus D98% divided by the median dose to the target volume (D50%) [17]. Lower values (closer to 0) indicate a better homogeneity. For the conformity index we used the formula proposed by Van’t Riet [18]. The conformity index reaches values between 0 and 1, whereas 1 would represent a reference isodose covering exactly the target volume and indicates optimal conformity. Gradient index was calculated by dividing the volume receiving 40.5 Gy (meaning the dose in between two dose plateaus of 30 and 51 Gy) by the volume of receiving the prescription isodose of 51 Gy. The lower the value of GI, the steeper the dose fall-off outside the target.

## Results

We identified a total of 63 patients treated with WBRT and simultaneous boost for brain metastases in the time from January 2015 and December 2020. Patients treated for limited (1–4) brain metastases, receiving HA-WBRT without SIB or receiving WBRT+SIB without HA have been excluded. In the end 12 patients treated with HA-WBRT+SIB for multiple brain metastases (> 4) have been identified. Median age was 58.5 (36 88) years and patients presented with a median number of 9 (6 to 17) metastases. The most common entity was NSCLC (n = 6), followed by breast cancer (n = 3). All patient characteristics are summarized in Table 1.

**Table 1:** Patient characteristics

Patient characteristics	
Age (years), median, range	58.5, 36–88
Gender (*n*), f/m	6/6
Primary tumor (n)	
Lung cancer	6
Breast cancer	3
Other	3
KPS at initial presentation, median, range	90, 60–100
PTV of metastases/resection cavities (ml) Median, range	19.0 (4.1–48.7)
PTV of whole brain (ml) Median, range	1724.5 (1465.4–2147.5)
Total hippocampal volume (ml) Median, range	3.5 (2.7−5.8)
Number of metastases (n), median, range	9. 6–17

**Fig. 2:**
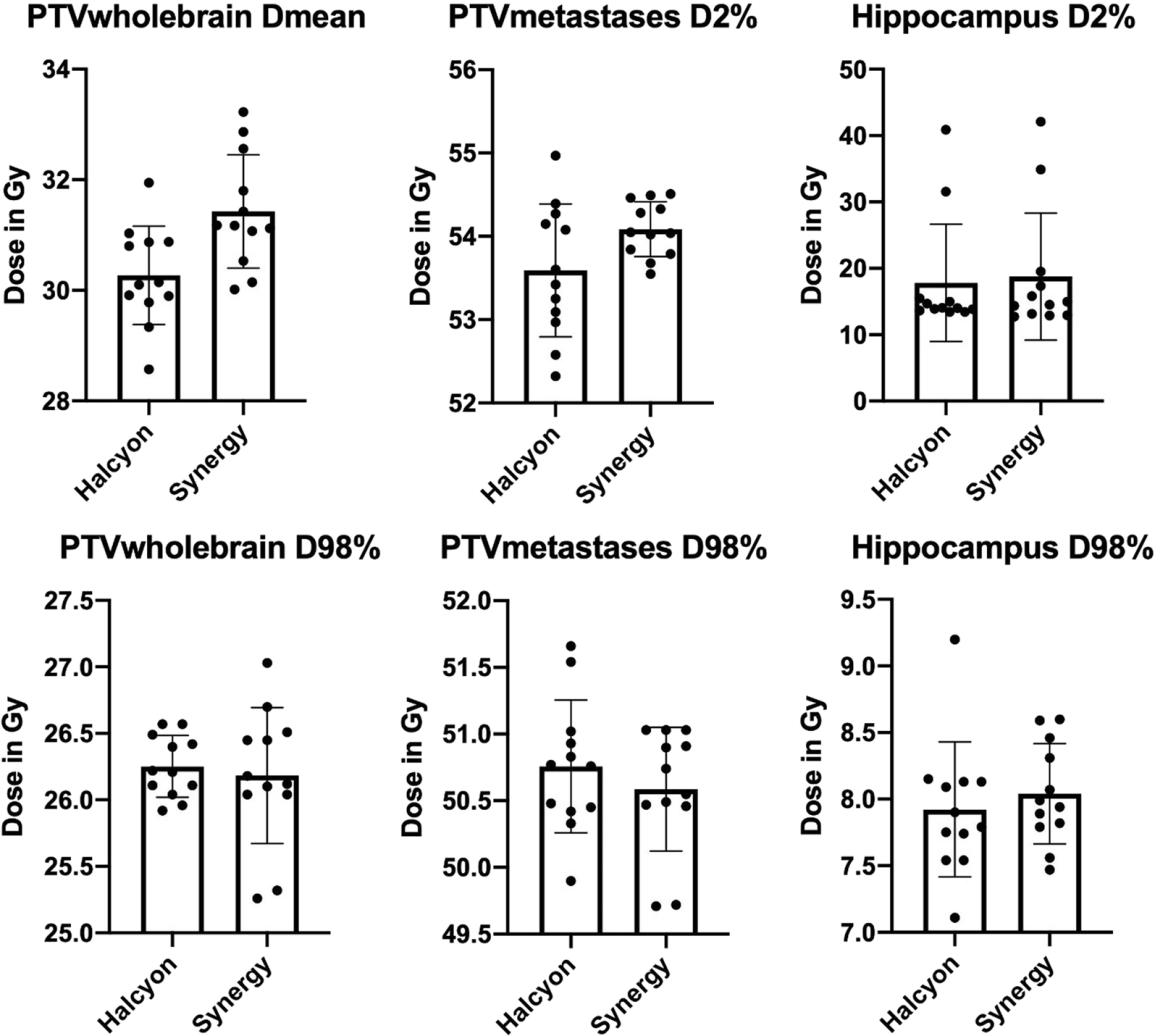
Box plots showing dose-values of target structures (PTVwholebrain, PTVmetastases) and Hippocampus

All treatment plans reached the goals for the target volumes (PTVwholebrain and PTVmetastases), see Table 2. Halcyon plans were significantly better in reaching the required Dmean of PTVwholebrain, although all Synergy plans reached values that were still acceptable according to the protocol (See Table 2). Dmax of PTVwholebrain was significantly less with the Halcyon treatment plans, albeit there was no specific protocol requirement. See Boxplots in Fig. 2.

**Table 2:** Study Protocol specifications and requirements for target structures and organs at risk

Structure (target/OAR)	Parameter and required value per protocol	Minor deviation per study protocol	Major deviation per study protocol	Halcyon (Eclipse)	Synergy (Pinnacle)	*p*-value	Number of major deviations (n)		Number of minor deviations (n)	
							H^**+**^	S^**#**^	H^**+**^	S^**#**^
PTVwholebrain	D98% ≥ 25 Gy	24 Gy ≤ D98% < 25 Gy	D98% < 24 Gy	26.25 ± 0.23	26.18 ± 0.51	0.71	0	0	0	0
	Dmean ≤ 35 Gy	35 Gy < Dmean ≤ 37 Gy	Dmean > 37 Gy	30.27 ± 0.89	31.43 ± 1.02	< 0.01	0	0	0	0
	Dmax	*	*	53.53 ± 0.85	54.67 ± 1.14	0.01	*	*	*	*
PTVmetastases	D98% ≥ 48.4 Gy	47.4 Gy ≤ D98% < 48.4 Gy	D98% < 47.4 Gy	50.76 ± 0.50	50.59 ± 0.46	0.07	0	0	0	0
	D2% ≤ 63.7 Gy	63.7 Gy < D2% ≤ 66.3 Gy	D2% > 66.3 Gy	53.59 ± 0.80	54.09 ± 0.33	0.07	0	0	0	0
	Dmean	*	*	52.28 ± 0.58	52.44 ± 0.26	0.25	*	*	*	*
	Dmax	*	*	54.96 ± 0.95	55.48 ± 0.54	0.09	*	*	*	*
GTVmetastases	Dmean	*	*	52.54 ± 0.71	52.74 ± 0.23	0.30	*	*	*	*
	Dmax	*	*	54.93 ± 0.99	55.45 ± 0.54	0.09	*	*	*	*
Hippocampus	D98% ≤ 9 Gy	9 Gy < D98% ≤ 10 Gy	D98% > 10 Gy	7.92 ± 0.51	8.04 ± 0.38	0.58	0	0	1	0
	D2% ≤ 17 Gy	17 Gy < D2% ≤ 18 Gy	D2% > 18 Gy	17.82 ± 8.84	18.77 ± 9.56	0.13	2	3	0	1
Optical nerves	D2% ≤ 33 Gy	33 Gy < D2% ≤ 35 Gy	D2% > 35 Gy	29.52 ± 7.41	28.62 ± 7.63	0.13	0	0	0	0
Chiasm	D2% ≤ 33 Gy	33 Gy < D2% ≤ 35 Gy	D2% > 35 Gy	30.20 ± 5.20	31.36 ± 5.67	0.15	1	1	0	1
Lenses	D2% ≤ 7 Gy	7 Gy < D2% ≤ 10 Gy	D2% > 10 Gy	5.65 ± 1.48	5.57 ± 0.71	0.91	0	0	1	0
Inner ear	D2% ≤ 33 Gy	33 Gy < D2% ≤ 35 Gy	D2% > 35 Gy	31.37 ± 6.60	32.59 ± 6.03	0.23	2	2	0	2
Eyes	D2% ≤ 33 Gy	33 Gy < D2% ≤ 35 Gy	D2% > 35 Gy	20.80 ± 4.35	18.16 ± 5.97	0.03	0	0	0	0
Brain stem	D2% ≤ 33 Gy	33 Gy < D2% ≤ 35 Gy	D2% > 35 Gy	34.02 ± 3.50	32.16 ± 3.38	0.01	3	2	1	1

Median values ± standard deviation (SD) for Halcyon (Eclipse) and Synergy (Pinnacle). Number of major and minor protocol deviations for H+ Halcyon (Eclipse) and S# Synergy (Pinnacle). *missing study protocol specifications

**Fig. 3:**
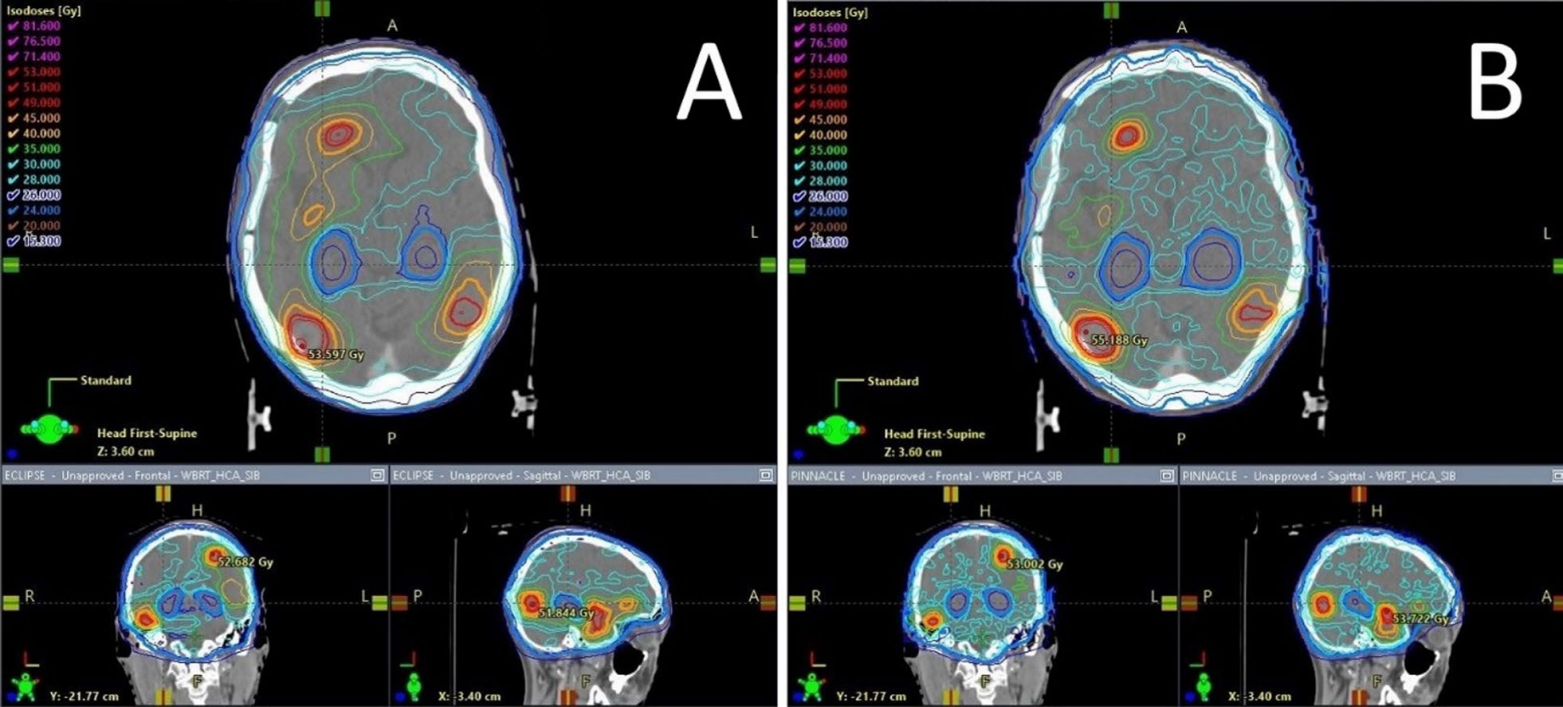
Dose distribution of HA-WBRT+SIB in Halcyon(Eclipse) and Synergy(Pinnacle) treatment plans

There were several protocol violations for organs at risk. In total we observed 8 major deviations and 3 minor deviations for the Halcyon treatment plans and 8 major and 5 minor deviations for the Synergy plans. The protocol criteria for D2% in the hippocampus wasn’t met four times with Synergy plans (3 major deviations, 1 minor deviation) and twice with Halcyon plans (2 major deviations). All other protocol violations can be seen in Table 2. A typical typical dose-distribution of both linacs in a representative layer is illustrated in Fig. 3.

**Fig. 4:**
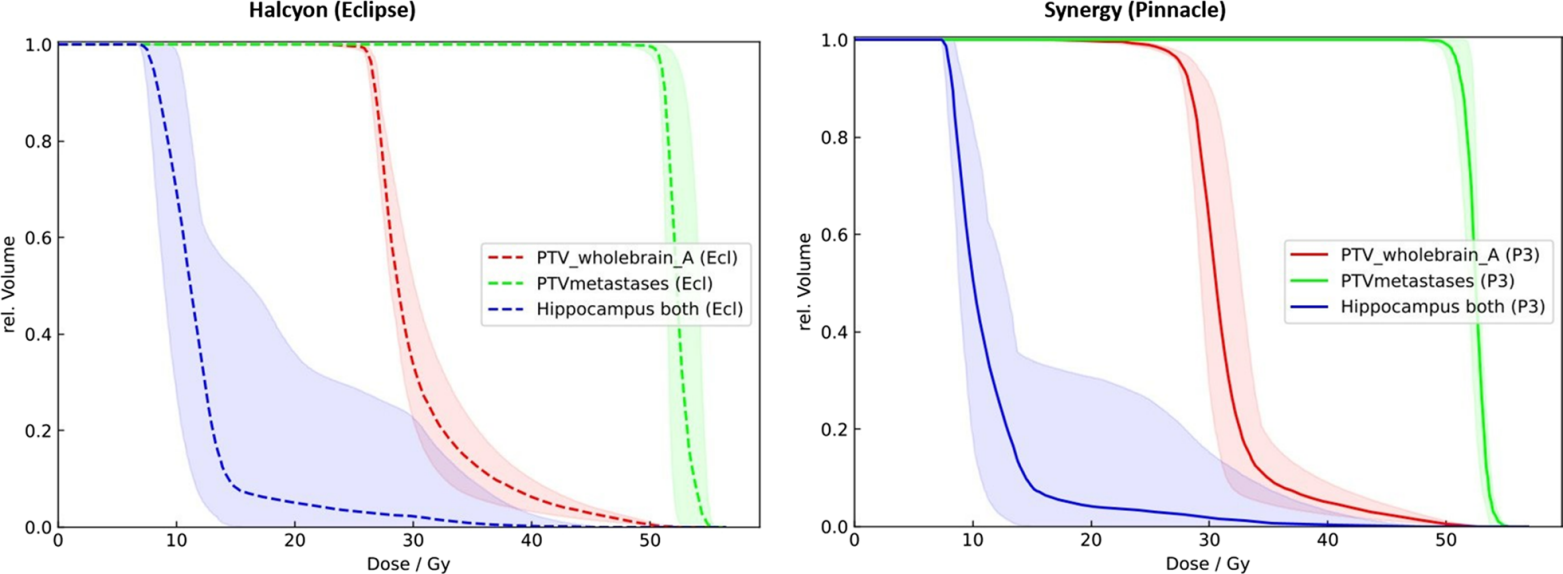
Average dose-volume histograms of PTVwholebrain, PTVmetastases and Hippocampus

Homogeneity Index for PTVwholebrain was superior in Synergy plans compared to Halcyon plans for both target structures (PTVwholebrain and PTV metastases) with 0.36 ± 0.05 versus 0.51 ± 0.04, respectively 0.04 ± 0.01 versus 0.02 ± 0.01. Conformity Index for PTVwholebrain was superior with Synergy plans with 0.58 ± 0.18 versus 0.30 ± 0.09 for Halcyon plans, but slightly more favourable with Halcyon plans for PTVmetastases with 0.69 ± 0.15 versus 0.68 ± 0.14. Gradient index was superior with Synergy plans 4.8 ± 21.68 versus 5.60 ± 1.95 for Halcyon plans. See Table 3 for an overview on HI, CI and GI. Average dose-volume histogram for both linacs is illustrated in Fig. 4.

**Table 3:** Values of Homogeneity Index (HI), Van’t Riet Conformity Index (CI) and Gradient Index (GI) for PTVwholebrain and PTVmetastases

	Index	Formula	Value Halcyon	Value Synergy
PTV_wholebrain	HI	$\frac{D2\% - D98\%}{D50\%}$	0.51 ± 0.04	0.36 ± 0.05
	CI	$\frac{TVri}{TV} x \frac{TVri}{Vri}$	0.30 ± 0.09	0.58 ± 0.18
PTVmetastases	HI	$\frac{D2\% - D98\%}{D50\%}$	0.04 ± 0.01	0.02 ± 0.01
	CI	$\frac{TVri}{TV} x \frac{TVri}{Vri}$	0.69 ± 0.15	0.68 ± 0.14
	GI	$\frac{V \text{40,5}}{V 51}$	5.60 ± 1.95	4.8 ± 21.68

## Discussion

Hippocampal avoidance radiotherapy was initially investigated in RTOG trial 0933 by Gondi et al. [13]. Its association with preservation of memory and quality of life was then further evaluated with the phase III randomized CC001 trial [14]. CC001 phase III trial randomized 518 patients to HA-WBRT plus memantine or conventional WBRT plus memantine. Primary endpoint was the time to cognitive function failure, defined as decline using a reliable change index in a large panel of neurocognitive tests. Deterioration in executive function at 4 months and learning and memory at 6 months was significantly better in HA-WBRT. In addition, patients receiving HA-WBRT and memantine reported less fatigue, less difficulty with remembering things and fewer cognitive symptoms as well as less difficulty with speaking. Besides promising results on hippocampal avoidance radiotherapy, other studies emphasized the value of dose escalation with simultaneous integrated boost to visible brain metastases compared to conventional WBRT which has been associated with improved local tumor control and even overall survival, at least in a subgroup presenting with limited brain metastases [19, 20]. To date, several data of non-randomized trials favor the integration of both - hippocampal avoidance as well as the integration of a concurrent boost [10, 21]. Both strategies, meaning dose escalation for improved local tumor control and preservation of cognitive function with hippocampal sparing, are fundamentally reflected in the concept of HA-WBRT+SIB. Nevertheless, the benefit of HA-WBRT has also been questioned in a recent study evaluating HA-WBRT in prophylactic irradiation of patients with small-cell lung cancer where a lower probability of cognitive decline could not be demonstrated [22]. Thus, premature conclusions for the optimal therapy in multiple brain metastases actually can not be drawn as data on randomized comparison of HA-WBRT with stereotactic radiotherapy in multiple brain metastases is still pending.

From technical point of view, requirements in HA-WBRT+SIB are demanding, since on the one hand dose escalation in the metastases has to be achieved, but in close proximity the dose should drop as low as possible in order to enable optimal sparing of the stem cells within the hippocampus.

In this study we compared the Halcyon O-ring linac/Eclipse TPS with a Synergy Agility C-arm linac/Pinnacle TPS for treatment of multiple brain metastases with hippocampal avoidance whole brain radiotherapy and simultaneous integrated boost. Plan performance was evaluated by using a dedicated study protocol of the HIPOORAD phase II study and adherence to the protocol requirements. The purpose of the study was to validate the ability of the new linear accelerator Halcyon to produce robust and reliable treatment plans for irradiation of multiple brain metastases with HA-WBRT+SIB and to therefore prove its suitability for treatment of brain metastases as its application in principle focuses on extracranial radiotherapy.

With regard to the results of our study—interpreted in the context of the required goals and constraints of the mentioned study protocol—we could confirm suitability of Varians new O-ring linac accelerator for the treatment of multiple brain metastases with HA-WBRT+SIB. All study protocol requirements for target structures (PTVwholebrain and PTVmetastases) were met by Halcyon plans as well as by Synergy plans. There was no statistical difference comparing the DVH-parameters of the study protocol except mean dose in PTVwholebrain, where Halcyon plans were significantly better. PTVwholebrain was prescribed with 30 Gy which in principle is intended to cover microscopic seeding of metastases. Although the optimal dose for the treatment of possible microscopic disease has not yet been clarified, overdose in PTVwholebrain and its healthy brain tissue should be avoided in any case, since chronic neurotoxicity might rather occur with an increasing dose [21, 23]. Plan quality values with HI, CI and GI might slightly favor Synergy plans, but those values haven’t been used as a quality criterion in the treatment planning process and are just reported for the sake of completeness.

We saw several violations of organs at risk structures, especially within the hippocampus and brainstem. We included two patients with brain metastases within the HAR (not inside the hippocampus) which explains the deviations within the required values for hippocampus. D2% in the hippocampus was better with Halcyon than with Synergy treatment plans, but without statistical significance. The violation of the brainstem constraints is reasoned by the closeness of some metastases to the brainstem. Brainstem was better spared with Synergy treatment plans (*p*-value = 0.01). We have to mention that several other patients formally did not meet the inclusion criteria of the HIPPORAD study as we did not restrict the total number of brain metastases (exceeding > 5 mm) to ten or did not exclude patients with large metastases (> 3.5 cm). We deliberately decided to leave these borderline cases in the study to test the two linacs in extreme scenarios as well and to keep a representative sample size in a real world cohort.

To date, there is no other study reporting on Halcyon`s performance for the treatment of HA-SIB+WBRT. In general there is only limited data and only two scientific papers reporting on the application of cerebral radiotherapy with the new linac accelerator [24, 25]. Of particular note is the impressive efficiency of irradiation of HA-WBRT with the Halcyon demonstrated by Yokohama et al. with a reduction in irradiation time of 1/9 compared to treatment with tomotherapy [25].

For treatment at the Synergy linac a dedicated hippocampal-blocking technique with a collimator angle of 85° has been used, where the field is splitted at the level of the hippocampi to allow optimal hippocampal sparing. Compared to conventional treatment planning, this technique was more effective in reducing the dose to the bilateral hippocampus in HA-WBRT+SIB [26]. The collimator setting with angles of 281°, 326°, 11° and 56° has been automatically suggested with VMAT planning in Eclipse with an integrated “arc-geometry tool”. Nevertheless, we did not find any significant difference between Eclipse and Halcyon plans in Hippocampus for D2% and D98% in compliance with the study protocol. RTOG 0933 and NRG Oncology CC001 trial suggested keeping 100% of the hippocampus less than 9 Gy. Mean value of D98% for both hippocampi has been 7.92 Gy for Eclipse plans and 8.04 Gy for Synergy plans and did not exceed 9 Gy except one case where a brain metastasis was located within the HAR. Beside the VMAT approach used in the underlying study other techniques exist for HA-WBRT, most notably IMRT and tomotherapy [9]. In general arc based delivery (VMAT) is faster than conventional IMRT and tomotherapy [25, 27].

A limitation of the O-ring accelerators is the impossibility for correcting rotational set-up errors. This fact might represent a limitation of those accelerators so far, although small rotational errors might not become relevant as a 1 mm margin is added to the brain metastases. However, larger rotational errors could have an impact on dose escalation in the metastases. Nevertheless, the dose will not fall below a therapeutic dose of 30 Gy which is prescribed to the surrounding PTVwholebrain, under the premise that the metastases are not located close to the hippocampal avoidance region. Devices to compensate for rotational errors in O-ring accelerators or tomotherapy are already under investigation and will certainly have an added value for HA-WBRT+SIB on O-ring linac accelerators without treatment couches with six degrees of freedom [28, 29].

A limitation of the underlying study is that two different treatment planning systems were used, each with different optimization algorithms. For every iteration in Pinnacle, the optimizer will use the gradient of the objective function with respect to the optimization parameters (leaf positions and weights) to find an update of the parameters that improves the objective function. Further differences are the beam quality which were used for treatment planning (6 MV FFF against 6 MV FF) and the different MLC design. Differences in the MLC design, beam quality and optimization algorithms of the two TPS/linac combinations may have introduced some uncertainties in the dose comparison.

In conclusion, both Halcyon as well as Synergy Agility linac accelerators produced clinically comparable treatment plans for hippocampal avoidance whole brain irradiation with integrated boost in patients with multiple brain metastases. These results indicate that the new Halcyon accelerator can be used for HA-WBRT + SIB according to the compliance criteria defined by a dedicated study protocol and suggest that the application of Varians Halcyon is definitely beyond extracerebral radiotherapy.

## Data Availability

We take full responsibility for the data, the analyses and interpretation, and the conduct of the research. We have full access to all of the data and provide data on request.
